# Emulsion Stabilization Strategies for Tailored Isocyanate Microcapsules

**DOI:** 10.3390/polym15020403

**Published:** 2023-01-12

**Authors:** Mónica V. Loureiro, António Mariquito, Mário Vale, João C. Bordado, Isabel Pinho, Ana C. Marques

**Affiliations:** 1 CERENA-Centro de Recursos Naturais e Ambiente, Departamento de Engenharia Química, Instituto Superior Técnico, Universidade de Lisboa, Avenida Rovisco Pais, 1049-001 Lisbon, Portugal; 2 CIPADE-Indústria e Investigação de Produtos Adesivos, SA. Av. Primeiro de Maio 121, 3700-227 São João da Madeira, Portugal

**Keywords:** microcapsules, emulsion, O/W, polyvinyl alcohol, interfacial polymerization

## Abstract

We report on the stabilization of an oil-in-water (O/W) emulsion to, combined with interfacial polymerization, produce core–shell polyurea microcapsules (MCs) containing isophorone diisocyanate (IPDI). These will act as crosslinkers for mono-component adhesives. The emulsion stabilization was evaluated using three types of stabilizers, a polysaccharide (gum arabic) emulsifier, a silicone surfactant (Dabco^®^DC193), a rheology modifier (polyvinyl alcohol), and their combinations. Emulsion sedimentation studies, optical microscopy observation, and scanning electron microscopy enabled us to assess the emulsions stability and droplet size distribution and correlate them to the MCs morphology. Fourier transform infrared spectroscopy and thermogravimetric analysis revealed the MCs composition and enabled us to evaluate the encapsulation yield. All stabilizers, except DC193, led to spherical, loose, and core–shelled MCs. The rheology modifier, which increases the continuous phase viscosity, reduces the emulsion droplets sedimentation, keeping their size constant during the MCs’ synthesis. This allowed us to obtain good quality MCs, with a smaller average diameter, of approximately 40.9 µm mode, a narrower size distribution and 46 wt% of encapsulated IPDI. We show the importance of the emulsion stability to tune the MCs morphology, size, and size distribution, which are critical for improved homogeneity and performance when used, e.g., in natural and synthetic adhesive formulations industry.

## 1. Introduction

Emulsions can be divided into simple emulsion systems, consisting of two immiscible liquids dispersed in one another or double/multiple emulsion systems consisting of three or more different fluids, as depicted in Scheme S1 from the Supporting Information [1,2]. Even if immiscible, the dispersion of two liquids in one another is thermodynamically unstable and the emulsion tends to break down over time. This can occur by gravitational separation due to sedimentation or creaming of the emulsion droplets by droplet aggregation or flocculation when two or more droplets associate with each other maintaining their individual integrity, and by Ostwald ripening due to mass diffusion from smaller to larger droplets [2,3,4,5,6]. All the referred mechanisms might lead to phase separation [2,4,5]. To overcome this tendency, it is possible to use different types of stabilizers, which include surfactants, amphiphilic polymers, and solid particles [3]. Surfactants, which can also include amphiphilic polymers, act by forming an electrostatic or steric barrier, decreasing attractive interaction between the emulsion droplets, and by reducing its interfacial tension facilitating the droplets disruption during the homogenization [1,4,5,6,7]. Regarding amphiphilic polymers, when adsorbed, there is a repulsive effect caused by the entanglement of polymer segments from two distinct droplets, since conformational rearrangements become limited due to their high molecular weight. Solid particles adsorb at the droplet’s surface to form a mechanical barrier against coalescence. An alternative strategy to the adsorption of stabilizers is the use of rheology or texture modifiers, such as thickening or gelling agents, which help to improve the kinetic stability of the system by increasing the viscosity or gel formation of the continuous phase, restricting the droplets movement [4,5].

For the O/W emulsion systems, the instability is typically a result of gravitational separation and flocculation, possibly leading to coalescence [3,5,7]. Liang et al., 2018, studied the effect of several variables on the stability of O/W emulsions, in particular the effect of the hydrophilic lipophilic balance (HLB) value of the surfactant system, its concentration, and the stirring time and intensity applied [7]. The authors concluded that the surfactant is the parameter which most stabilizes the emulsion, with its stabilizing effect depending on the combination of droplet interface film thickness, interfacial tension, and molecular structure [7]. Wang et al., 2020, reported on three natural polysaccharides, officinale polysaccharide (DOP), propylene glycol alginate (PGA), and gum arabic (GA), and combinations between them, for the stabilization of an O/W emulsion system [8]. GA is the polysaccharide that most reduced the emulsion interfacial tension, and the size of the droplets, when compared to PGA, due to its good adsorption rate. However, the smallest ones were obtained with DOP due to its high viscosity [8]. 

GA is one of the most used emulsion stabilizers for the synthesis of polyurethane/polyurea (PU/PUa) MCs obtained by interfacial polymerization combined with an O/W emulsion system due to its good water solubility, low solution viscosity, good surface activity, and ability to form a protective film around emulsion droplets [8,9,10]. However, GA is composed of arabinogalactan (88.4%), arabinogalactan protein (10.4%), and glycoprotein (1.24%), and its emulsifying property is mainly attributed to arabinogalactan protein, which is the reason for the high amounts needed to reach a stable emulsion [8,9]. Studies report on the optimization of the GA concentration targeting the control of the MCs’ morphology, size, and size distribution [10,11,12]. It was observed that as the GA concentration increases, the sphericity and uniformity of the MCs improve. However, it is only for concentrations of GA above 12 wt% that a significant reduction in the MCs size occurs, which is correlated with a considerable increase in the continuous phase viscosity [10,11,12]. Examples of other stabilizers reported in the literature for this application are Tween 80, Tween 20, Sodium dodecyl sulfate (SDS), SPAN20, polyvinyl alcohol (PVA), dodecyl benzene sulfonate (SDBS), and combinations of GA with CTAB (cetrimonium bromide) and PVA with SDS [11,12,13,14]. As an alternative, microfluidic systems have also been used to produce MCs with homogeneous sizes; however, batch processes are better implemented in the industry and allow for a higher production output [15,16,17].

We have previously reported on the synthesis of spherical and loose PU/PUa MCs containing encapsulated isocyanate, monomeric and oligomeric species, either composed by a PCL or PU/PUa shell [18,19,20,21,22]. The emulsion of the MCs prepared by interfacial polymerization technique, either by batch or by a continuous method using a custom-made microfluidic system, were stabilized using GA [18,19,20,21]. Our current purpose is to reduce their size and size distribution, but mainly to understand the impact of the emulsion stability on the control of the MCs final morphology.

The developed MCs are intended to substitute the current crosslinkers used in PU and polychloroprene (PCP) adhesives which are currently supplied as two-component (2K) formulations composed of a polyol OH prepolymer and a crosslinker, usually isocyanate-based [23,24,25,26]. The microencapsulation of the isocyanate reduces or eliminates the risk associated with its handling, and at the same time offers control over its triggered release. The MCs respond to the stimuli of pressure and/or temperature applied during the adhesive joint preparation, which leads to the isocyanate release and cure of the adhesive. Although the developed MCs already proved to be efficient crosslinkers for this application, reported at Attaei et al., 2018, a smaller size distribution would lead to a more homogeneous and reproducible adhesive joint, as different sized MCs might contain different amounts of encapsulated content [18]. By an improved stability of the emulsion, it is possible to have better control over the synthesis and the characteristics of the MCs.

The innovation of this work relies on the fine-tuning of the PU/PUa final MCs’ size and size distribution, obtained by interfacial polymerization technique, by developing an efficient O/W emulsions stabilization system. For that, the effect of a polysaccharide, a surfactant and a polymer, and combinations between them were studied. GA, the polysaccharide, was the stabilizer used in previous studies and is here used as reference [18,19,20,21]. Partially hydrolyzed PVA, a non-toxic and biodegradable water-soluble polymer, is used to function as a thickening agent [27]. DC193 is a non-ionic silicone surfactant and was chosen based on its HLB value, its low surface tension, and its use in the PU and footwear industry. The stabilization system is intended to enable: (i) the production of a stable emulsion with a small size distribution; (ii) a minimum variation between the size of the initial emulsion droplets and that of the final MCs, which will enable the control of the MCs’ final morphology; (iii) the production of MCs with a good encapsulation efficiency and shelf-life.

## 2. Materials and Methods

### 2.1. Materials

The shell-forming isocyanate, a commercial methylene diphenyl diisocyanate (MDI) oligomer, Ongronat^®^ 2500, and the monomeric IPDI, Desmodur^®^ I, to be encapsulated, were kindly supplied by BorsodChem (Kazincbarcika, Hungary) and by Covestro AG (Leverkusen, Germany), respectively. The active (hydrogen) H source Jeffamine^®^ D-2000, a polyetheramine, was supplied by Huntsman (The Woodlands, TX, USA). Regarding the emulsion stabilizers, the gum arabic (GA) was obtained from Fisher Chemical (Porto Salvo, Portugal), the Dabco^®^ DC193 was purchased from Air Products, Dow Corning (Bristol, PA, USA), and the Polyvinyl alcohol (PVA), (98–99% hydrolyzed, of medium molecular weight (MW), with an average of 57,000 to 66,000 gmol^−1^) was obtained from Alfa Aesar (Haverhill, MA, USA). All the chemicals were used as received, without further purification.

### 2.2. Method

#### 2.2.1. Oil-in-Water Emulsion

For the O/W emulsion formation, the oil and water phases were vigorously mixed at 3200 rpm using an Ultra-Turrax (IKA T25 digital ULTRA TURRAX, Germany), during 10 min, at 70 °C. The oil phase, which forms ca. 7 wt% of the emulsion, was composed by 30 wt% of the shell-forming MDI oligomer, Ongronat^®^ 2500, and 70% of Desmodur^®^ I. GA, DC193 and PVA, were the tested stabilizers (see Table S1 in the Supporting Information). Their concentration in the water phase varied according to Table 1. The emulsification conditions were maintained at constant for all the emulsions, varying only the stabilizer to assess its effect.

#### 2.2.2. In Situ Microcapsules Synthesis

MCs were synthetized using the previously prepared O/W emulsions, combined with in situ polymerization at the O/W interface. The synthesis procedure and the respective parameters used in this work are an adaptation from the processes developed by our group, described in Attaei et al., 2018, and Loureiro et al., 2020, with the schematic representation illustrated in the Scheme S2 from the Supporting Information [18,19].

An aqueous solution of Jeffamine^®^ D-2000 at 17 wt% is added to the synthesis to act as an active H source, making 2.7 wt% of the total emulsion system, while under a mild agitation of 400 rpm by using a mechanical stirrer, Heidolph RZR 2051 control (Heidolph Instruments, Schwabach, Germany), at 70°C. The temperature of the synthesis was measured using a laboratory thermometer placed inside the reactor. After 3 h, the MCs were filtrated using a vacuum filtration system, composed by a kitasato, a Büchner funnel, and a diaphragm pump model MD 12 NT from Vacuubrand (Wertheim, Germany). The MCs were washed with water during the filtration procedure and were left to dry at room temperature for 48 h before storage.

#### 2.2.3. Characterization

The emulsion was characterized by optical microscopy and optical observation. For the MCs characterization, optical microscopy, scanning electron microscopy (SEM), Fourier transform infrared spectroscopy (FTIR) with attenuated total reflectance (ATR) accessory, thermogravimetric analysis (TGA), and first-derivative curves (DTG) were used.

*Optical Microscopy*. A Kruss MSZ 5600 optical microscope (Hamburg, Germany) was used to evaluate the emulsion stability and droplets’ size variation over time, as well as the MCs’ shell maturity and stiffness during the synthesis. The emulsions in study were evaluated both while at rest and during the first 30 min of the MCs’ synthesis, as after that time it was not possible for us to guarantee that no solid film had been formed at the O/W emulsion interface. The average diameter and size distribution of the emulsion droplets were evaluated using photographs obtained by optical microscopy, in transmission mode, by employing the Fiji software in a sample of 100 droplets [27].

**Figure 1:**
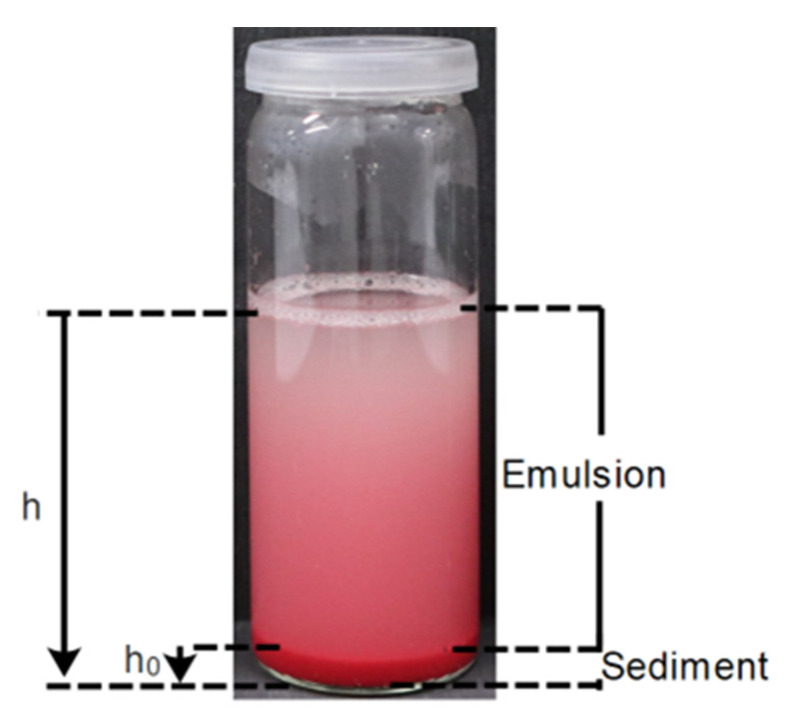
Visual explanation of the h and h_0_ used for Equation (1).

*Emulsion stability—Sedimentation tests*. An emulsion of each type was prepared using an organic dye, Oil Red O dye (Sigma Aldrich, St. Louis, MO, USA) in the oil phase, to better distinguish both phases. After the emulsion preparation, a 30 mL sample was collected in a vessel of 2.6 cm of diameter by 7.7 cm of height. The emulsion was followed for 30 min, while at rest. Digital photographs were taken at periodic intervals of 1 min, during the first 5 min, and then every 2 min. The following equation was used to calculate the sedimentation volume fraction of the emulsion during gravitational separation.

(1) $$Sedimentation=\frac{\pi \times r^{2}\times h_{0}}{\pi \times r^{2}\times h}$$

where *h* is the total height of the initial emulsion in the vessel, *h*_0_ is the height of the sediment formed at the bottom, as depicted in Figure 1, and *r* is the radius of the vessel used to calculate the volume.

*Scanning Electron Microscopy (SEM) and Energy-dispersive X-ray spectroscopy (EDS)*. The morphology, roughness, porosity, and size distribution of the MCs were assessed using photomicrographs obtained from an SEM equipment (Hitachi S2400, Chiyoda, Tokyo, Japan). All the photomicrographs were obtained in the secondary electron mode, always using the same SEM equipment. EDS data were obtained using a Phenom ProX G6 benchtop SEM (ThermoScientific, Waltham, MA, USA). The samples were immobilized in a sample holder using a conductive double-sided adhesive carbon tape and coated with a conductive 15 nm layer of gold–palladium thin film, through sputtering, by using a Quorum Technologies sputter coater, model Q150T ES (Lewes Road, Laughton, UK). The average diameter, size distribution, and shell thickness of the MCs were evaluated by using photomicrographs obtained through SEM, employing the Fiji software in a sample of 100 MCs [28]. Debris and non-spherical MCs were discarded in the calculations. 

*Viscosity*. The viscosity of the emulsions water phase was measured using a BROOKFIELD DV-II+Pro digital viscosimeter, with a CFE—52 spindle, at a speed of 60 rpm and a temperature of 20 °C. The measurements were carried out in triplicate.

*Fourier Transform Infrared Spectroscopy (FTIR)*. FTIR spectroscopy was used to assess the relative encapsulation yield and to confirm the chemical composition of the MCs’ shell. FTIR-ATR spectra of the MCs, isocyanate compounds, active H source and emulsion stabilizers were obtained for such purpose. The FTIR equipment used was a Spectrum Two from PerkinElmer (Waltham, MA, USA) equipped with a UATR Two accessory. The spectra were obtained at 4 cm^−1^ resolution and 8 scans of data accumulation.

Equation (2) was used to determine the relative encapsulation yield (Y value) of the MCs, which represents an indirect measure of the isocyanate encapsulation efficiency. For that, we considered the area of the peak related to free isocyanate (NCO group), at 2260 cm^−1^, and the area of a peak related to the PU/PUa shell, 1300 cm^−1^, that did not tend to significantly change over time. The FTIR peaks areas were obtained by using the Origin Pro 2016 software.

(2) $$Y=\frac{Area_{NCO\;\left(2600{\;\mathrm{cm}}^{-1}\right)\;}}{Area_{shell\;\left(1300{\;\mathrm{cm}}^{-1}\right)}}$$

where *Y* is considered a relative, indirect measured value of the isocyanate encapsulation efficiency, Area NCO (2600 cm^−1^) the area of the isocyanate’s NCO peak, and Area shell (1300 cm^−1^) the area of the C-O stretching peak, related to the MCs’ shell material.

*Thermogravimetric Analysis (TGA)*. TGA resulting thermograms and derivative curves (DTG) enabled do conclude about the amount of encapsulated isocyanate and the MCs’ shell composition, corroborating the FTIR analysis results. TGA was performed using a HITACHI STA 7200 Thermal Analysis System equipment (Ibaraki, Japan), under a controlled nitrogen atmosphere with a flow of 200 mL/min, at a temperature increase rate of 10 °C min^−1^, in the range of 30 to 600 °C.

## 3. Results

A large number of emulsions were prepared and studied using various types of stabilizers at different contents and combinations among them. The best five emulsion systems which represented different strategies were selected to be reported in this paper. A general overview for all cases can be found at Table S2 from the Supporting Information.

GA is the stabilizer we previously reported to obtain PU/PUa MCs by interfacial polymerization [18,19,20,21]. DC193 was used at 2.5, 4, 5, and 6 wt%, with the first leading to a complete coalescence of the emulsion and 5 and 6 wt% leading to large dimension MCs with mean diameters of 130 and 250 µm, respectively. Two different combinations of GA and DC193 were tested, both resulting in satisfactory MCs, with the combination here described leading to a narrower size distribution. PVA was used at 2 and 3 wt% based on previous studies. Finally, two combinations of PVA with GA were tested, with the selected one leading to smaller sized MCs and a narrower size distribution. SPAN^®^20, Tween^®^85 and Pluronic^®^ P-123 were also tested, at several percentages, resulting in a complete or partial destabilization of the emulsion.

Gravitational separation is one of the most common emulsion instability mechanisms when the two phases have a significant density disparity [1]. Although creaming is the most common phenomenon in O/W emulsions, Ongronat^®^2500 (1.24 g/mL, at 25°C) greatly contributes to increasing the oil phase density, so that the droplets have a higher density than the surrounding liquid (1.114 g/cm^3^ versus 0.997 g/cm^3^), and consequently a tendency to move downwards, which is referred to as sedimentation [1,29]. Such gravitational separation can be retarded by reducing the density difference between the water and oil phases, by increasing the viscosity of the continuous phase, or by decreasing the size of the droplets [1].

Our emulsions are polydisperse in terms of droplet size distribution, and therefore the droplets tend to sediment at different velocities, with the larger ones suffering from a faster sedimentation, as depicted in Scheme S3 from the Supporting Information. The smaller droplets tend to remain dispersed in the upper part of the emulsion during a prolonged time, and so it is not possible to distinguish a serum layer, composed solely of a continuous phase, during the time of the study. For this reason, we used the volume percentage of sediment (sedimentation layer) to draw conclusions regarding its stability over time. 

**Figure 2:**
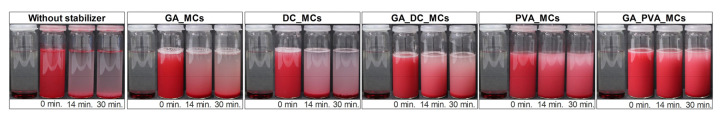
Visual observation of the emulsions used to obtain the GA, DC, GA_DC, PVA, and GA_PVA MCs, over a period of 30 min. From left to right: oil and water phase before emulsification, right after emulsification, 14 and 30 min after emulsification (static conditions).

**Figure 3:**
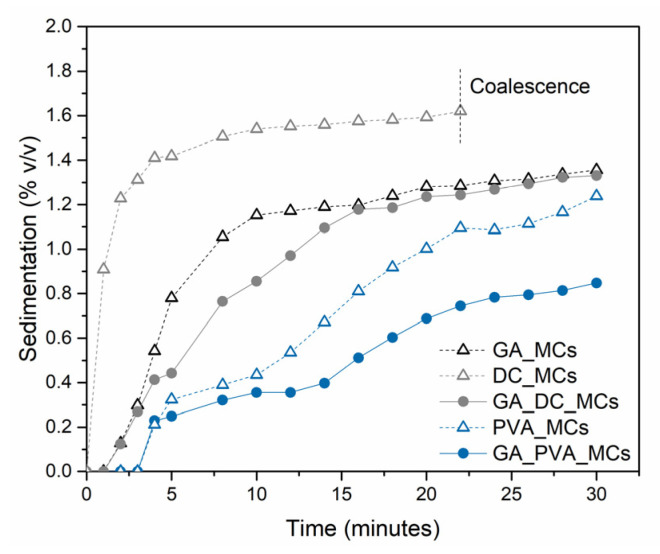
Evolution of the sedimentation volume fraction over time, of all the reported emulsions in study, while at rest (static conditions).

Figure 2 shows the photographs of the emulsions, 0, 15, and 30 min after the emulsification, while Figure 3 represents the evolution of the volume percentage of sediment along time, calculated using Equation (1). 

DC193 leads to the highest sedimentation volume fraction, of 1.6%, followed by GA, of 1.4%. The emulsion stabilized by DC193 is the only one suffering from coalescence, which may contribute to a quicker sedimentation due to the increased size of the droplets. This might occur due to the small size of the DC193 molecule, compared to the other stabilizers in the study, which makes it more difficult to avoid the attractive interactions between the isocyanate droplets [30]. The combination of GA and DC193 led to some improvement in the emulsion stability when compared with the two stabilizers acting alone. PVA led to significant improvements in decreasing the sedimentation, as it stabilizes the emulsion by rheology modification, increasing the viscosity of the continuous phase to 5.78 cP, and to 7.64 cP when in combination with GA, which significantly slows down the gravitational separation phenomena in emulsions [30,31].

After 30 min at rest, the density of droplets at the DC193 emulsion phase is significantly less than that of the GA_PVA emulsion, and the occurrence of coalescence in the sediment phase is notorious, revealing some inadequacy of the DC193 for this application (see Figure S1 from Supporting Information).

All the emulsions were left to rest for 72 h and, with the exception of the DC and GA_DC_MCs’ emulsions, it was possible to obtain loose, spherical MCs (see Figure S2 from the Supporting Information). 

The emulsions were also followed by optical microscopy during the first 25 min of the MCs´ synthesis procedure (dynamic conditions). As was observed for static conditions, the emulsion stabilized by DC193 was the only one suffering from coalescence, with no major changes reported with the other stabilizing systems (see Figure S3 from the Supporting Information).

**Figure 4:**
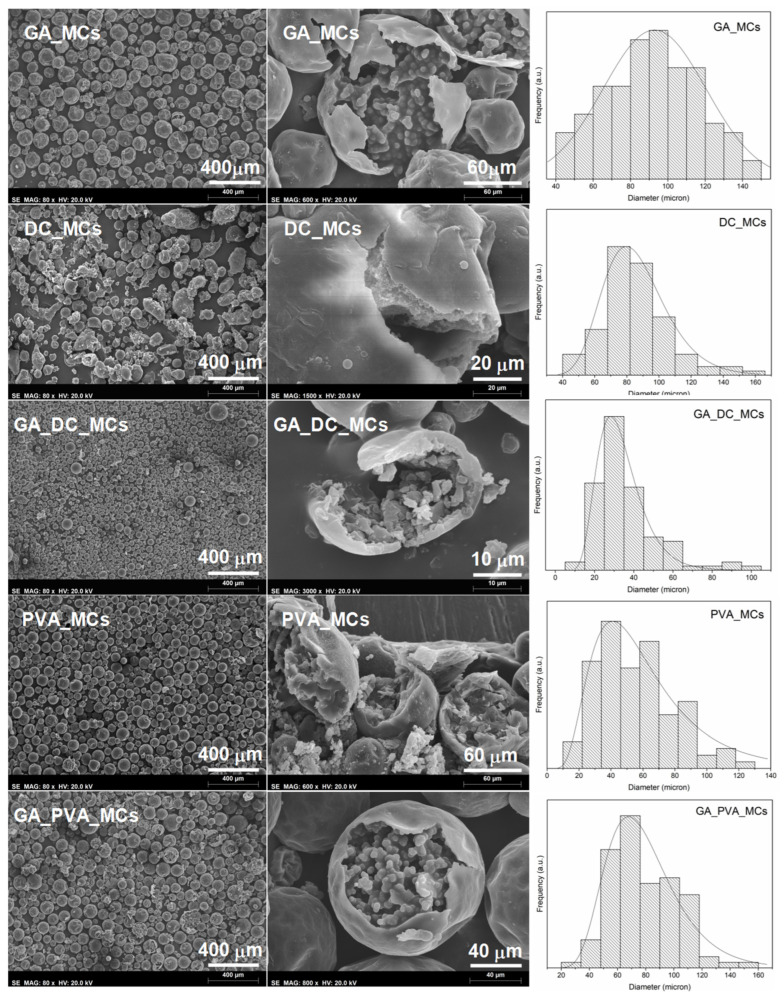
SEM images of the obtained MCs at 80x amplification (at the left). SEM images of isolated MCs’ cross sections, deliberately crushed (obtained at different magnifications). MCs’ size distribution (at the right).

All the stabilization systems in study led to loose MCs, with a core–shell morphology (Figure 4) and a spherical shape, except for DC193 which led to some irregular-shaped MCs and debris. The MCs show some roughness, or buckling, which is usually attributed to fluid-induced shear forces related to the mechanical stirring during the synthesis, and the inhomogeneous formation of the MCs shell due to different reaction kinetics of the isocyanate with the active H sources and water. A slow shell formation, which leads to an initially thin and flexible shell that precedes the reduction of the MCs size, due to the progress of the interfacial polymerization reactions, may also contribute to this effect. [32,33,34]. 

The combining effect of GA and DC193 (at a 4:1 ratio) led to the smallest MCs. We believe that both the strong steric effect of the GA, due to the entropy caused by the GA branched polysaccharides hydrophilic chains, and the low surface tension provided by the DC193 surfactant contributed to the emulsion stabilization. It has been stated that when the two stabilizers coexist and reach an equilibrium state, the silicone surfactant preferentially migrates to the emulsion droplets interface and, only when this is at low concentrations does the GA partially adsorb [35]. Indeed, a DC193/GA ratio of ca. 1:1 was also tested, leading to bigger MCs with a broader size distribution.

We have also studied the effect of Tween^®^85 and SPAN^®^20, both hydrocarbon chain surfactants and, for both cases, the emulsion was lost while under mechanical agitation (see images in Table S2 from the Supporting Information). This is possibly due to their lower HLB values of 11 and 9, respectively, compared to that of DC193, which gives them a lower affinity for the water phase of the O/W emulsion. [36,37,38].

All the samples follow a monomodal distribution (Table 2) with GA_MCs having the broadest profile. On the other hand, the combination of DC193 and GA led to the narrowest size distribution, with most of the GA_DC_MCs being between 14.8 and 45 µm in diameter. It is important to state that no debris or non-spherical MCs were considered for the size distribution determination, which is particularly relevant for the DC_MCs. 

PVA was found to greatly increase the viscosity of its aqueous solutions. Indeed, by increasing the viscosity of the continuous phase it is possible to reduce the collisions between the O droplets and their tendency for sedimentation. The latter one also tends to increase by the density disparity between the O and W phases [39,40,41]. When added at contents as low as 2 wt%, it leads to a viscosity of 5.8 cP, which is significantly higher than that obtained for GA at 5 wt%, namely 1.3 cP. PVA is, therefore, a more efficient rheology modifier, which plays an important role in emulsion stabilization and results in smaller MCs with a narrower size distribution. Larger contents of PVA were studied and found to lead to bigger sized MCs and a larger size distribution, which might be associated with an excessive viscosity of the continuous phase. The same has occurred to the GA_PVA emulsion, with a viscosity of 7.6 cP, resulting in larger MCs when compared to the PVA_MCs. Moreover, it has been reported that excessive free, non-adsorbed, polysaccharide emulsifiers on the continuous phase of O/W emulsions can lead to depletion-induced flocculation, promoting aggregation, creaming, and finally coalescence of the emulsion droplets [42,43]. In addition, the higher viscosity of the aqueous phase of the GA_PVA emulsion requires more energy to break the oil phase into smaller droplets and hinders the diffusion of GA and adsorption onto the oil droplets, contributing to a less-stable emulsion [42,43]. We suggest here that there is an optimum viscosity value for the continuous phase to lead to a stable emulsion, namely 5-6 cP for the present system.

The MCs’ diameter (D), average shell thickness (S), and ratio between the two are listed in Table 2. Smaller sized emulsion droplets have more surface area and, consequently, there is more contact between the isocyanate species and the aqueous phase, which can increase the PU/PUa shell fraction. In addition to its usually lower core content, smaller MCs are harder to break by the effect of pressure applied during the preparation of the adhesive joint, which may compromise the isocyanate release. For this reason, we considered a mode of 35 µm size as the minimum acceptable for the envisaged application. 

GA and GA_PVA_MCs are the ones exhibiting the lowest S/D ratio, indicating a smaller shell contribution to the MCs morphology, while GA_DC_MCs are the smallest MCs.

**Figure 5:**
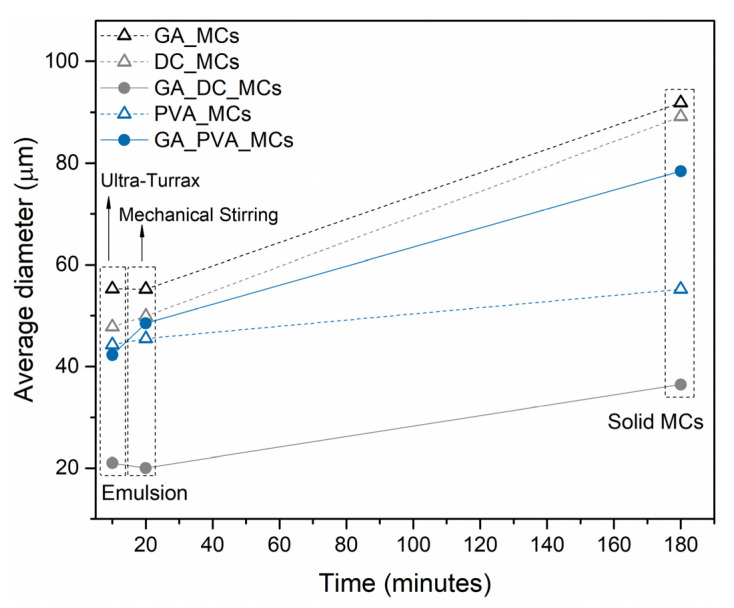
Oil emulsion droplets average diameter at the end of the emulsion preparation (10 min), after 10 min under mechanical agitation (20 min), and of the final MCs (180 min).

Figure 5 represents the average diameter of the oil emulsion droplets at the end of the emulsification process, using the Ultra-Turrax, after 10 min under mechanical agitation and of the final MCs. The diameters of the oil droplets at the end of the emulsification process correspond to those reported for the emulsion before the MCs synthesis, labeled in Figure 5 as “Ultra-Turrax”. The more stable the emulsion is at the initial stage of the synthesis, the less likely it is to suffer variations over time until it reaches the solid MC form. DC193 led to the largest variation between the initial emulsion and the final MCs’ size, even when excluding the debris and non-spherical particles, followed by GA. GA_PVA_MCs suffered a final variation identical to the one obtained with GA, however with a more noticeable destabilization in the first 10 min. PVA_MCs suffered the lowest variation, which proves the PVA capability to stabilize O/W emulsions, until the solid MC stage is reached, offering the possibility of a finer tuning of the achieved MCs. By using PVA as the emulsion stabilizer, it will be possible to define the MCs size by controlling the size of the initial emulsion droplets. If smaller MCs are preferable, it is possible to decrease the emulsion size using a higher emulsification rate.

**Figure 6:**
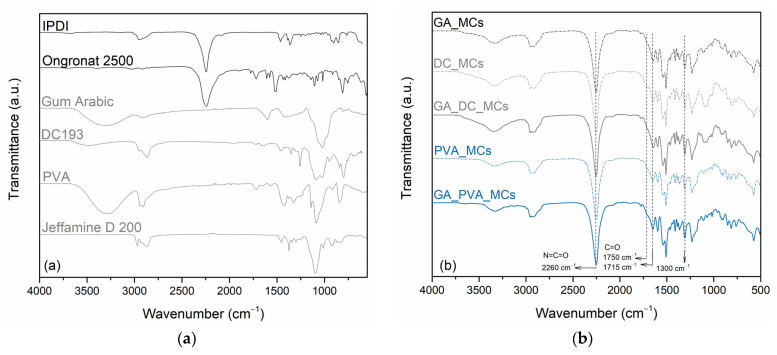
FTIR spectra of the isocyanates, active H source, and stabilizers (**a**). FTIR spectra of the MCs (**b**).

ATR-FTIR spectra of the MCs are presented in Figure 6, along with those of the isocyanates, active H source, and stabilizers. The presence of a peak at 2260 cm^−1^ in the MCs FTIR spectra, related to the presence of a N=C=O bond stretching vibration from unreacted isocyanate, confirms its encapsulation, i.e., its presence in the MCs’ core [42,43]. We conclude that IPDI is the encapsulated isocyanate by the similarity of the MCs’ and the raw material isocyanates´ NCO peak shapes. The presence of PU and PUa, from the MCs’ shell composition, is revealed by the peaks at 1780–1760 cm^−1^ and at 1760–1700 cm^−1^, which are related to the carbonyl groups from urethane and urea linkages, respectively, in addition to the peak at 1214 cm^−1^ from the presence of C–O–C group and the peak at 1300 cm^−1^ from the stretching of C–O linkages [44,45]. PUa carbonyl groups are clearly detected in all the MCs spectra while the ones from PU are only slightly detected. DC_MCs are the ones which exhibit a stronger PU carbonyl peak, which suggests that DC193 OH end groups reacted with the Ongronat^®^ 2500 to a higher extent than the other stabilizers. Indeed, a broad band between 1115 cm^−1^ and 1060 cm^−1^ in the DC_MCs and GA_DC_MCs spectra indicate the presence of siloxane (Si-O-Si) groups and Si-O-C bonds brought by DC193 [46]. An additional EDS analysis confirmed the presence of Si atoms in the DC_MCs (see Figure S4 and Table S3 from the Supporting Information). The presence of GA and PVA characteristic peaks cannot be identified or are hidden in the respective MCs spectra.

To study the MCs’ shelf-life, we calculated the Y value right after the MCs’ synthesis (Yi) and 3 months afterwards, with the latter given as a percentage of Yi. The obtained values are reported in Table 3. We can state that GA_PVA_MCs and PVA_MCs are the ones with the highest encapsulation value, while DC_MCs is the sample with the lowest one. In fact, for the latter, there is evidence of DC193 incorporation in the MCs’ shell, which is in accordance with the lowest Y value. PVA is the stabilizer that is expected to have a lower contribution to the MCs’ shell composition as it does not act at the emulsion droplet interface, it stabilizes the emulsion only by increasing the viscosity of the water phase, which might explain the larger encapsulation observed. GA, on the contrary, forms a dense network around the oil droplets, which might hinder the interfacial polymerization to some extent. Indeed, the GA_MCs needed almost 30 min more to mature than the PVA_MCs, which might have influenced the encapsulated content. 

By comparing the Y (%) values obtained after 3 months, it is possible to draw conclusions about the MCs’ shelf-life, directly related to the shell permeability to moisture. If the MCs’ shell does not offer enough protection, moisture can diffuse to the interior of the MCs and react with the free NCO groups, leading to a loss of encapsulated compound, together with an increase in the shell’s thickness and a decrease in the Y value. During the 3-month period, all the MCs except for the GA_DC_MCs showed a decrease in the Y value between 37.7 and 54.2%. GA_DC_MCs are substantially smaller than the remaining samples, therefore, with a larger surface area which contributes to a greater diffusion of air moisture to the core and consequent isocyanate polymerization, this explains the almost 66% decrease in the Y value.

**Figure 7:**
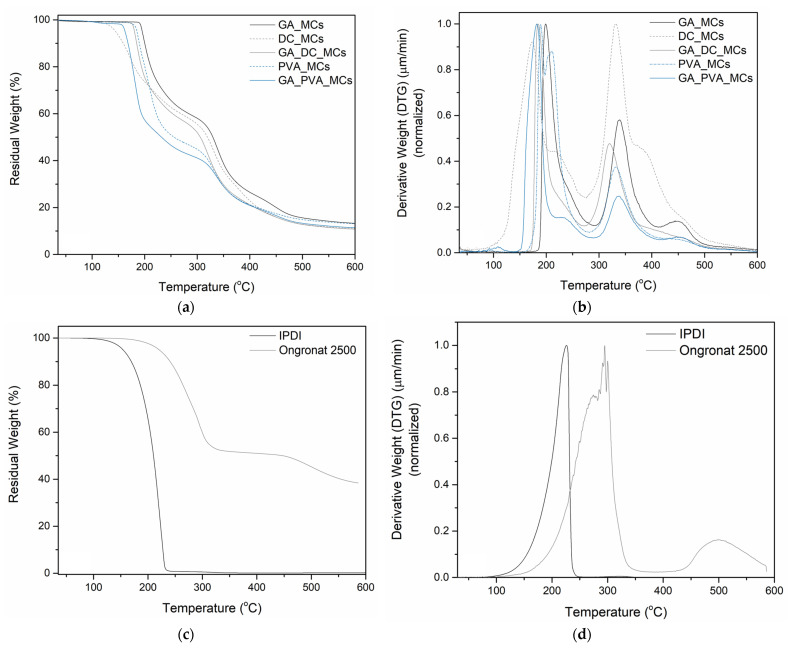
Thermogram of the as-prepared MCs (**a**) and the respective derivative curves (**b**). Thermogram of the isocyanates (**c**) and the respective derivative curves (**d**).

Thermogravimetric analysis was used to quantify the encapsulated isocyanate and to corroborate the findings obtained by FTIR-ATR. The thermograms and respective derivative curves of the MCs, as well as those of the isocyanates used in the synthesis, are represented in Figure 7. The first thermal event occurring to Desmodur^®^I starts above 100 °C, with the major mass loss occurring at 225 °C, respecting its evaporation, while the Ongronat^®^2500 has its most significant loss occurring at 294 °C. The PUa and PU MCs’ shell decomposition, especially the soft segments, start to occur typically around 300 °C (see Figure S5 from Supporting Information) [20]. Taking this into account, we considered the first slope of the MCs thermograms to calculate the amount of encapsulated isocyanate (Table 3). All the MCs have around 30 wt% of unreacted encapsulated isocyanate, with the exception of the PVA_MCs and GA_PVA_MCs, both having ca. 46 wt%. As stated above, PVA leads to a better interaction between the aqueous and oil phase, leading to a quicker PUa barrier formation and consequently to a higher encapsulation yield.

## 4. Conclusions

This work reports on the stabilization of an O/W emulsion system by interfacial polymerization, intended to be used in the synthesis of PUa shell MCs. The MCs are envisaged as crosslinkers for mono-component adhesives, an application for which small sized MCs, with a minimum diameter of 35 µm, and a narrow size distribution, allow a better adhesive performance.

Three different types of stabilizers, and combinations between them, are herein presented for the emulsion stabilization, in particular a polysaccharide biopolymer (GA), a surfactant (DC193), and a rheology modifier (PVA). PVA and its combination with GA led to a significant reduction in the gravitational separation phenomenon, i.e., reduction in the oil phase sedimentation, with the continuous phase viscosity having the most significant impact on the emulsion stabilization. The MCs obtained with the combination of both stabilizers were also the ones with the highest encapsulation content (46.4 wt%). However, PVA alone, besides leading to a similar encapsulation amount, showed a better performance during the MCs’ synthesis, leading to the smallest variation in the size of the droplet-to-particle process, and allowing a finer tuning and control of the MCs’ size. Using PVA as an emulsion stabilizer allowed us to define the MCs size by controlling the size of the initial emulsion droplets.

All the stabilizers enabled loose, spherical, and core–shelled MCs, with the exception of DC193, which led to some irregular-shaped MCs and debris. In particular, the use of PVA and the combination of GA with DC193 led to small-sized MCs, with the size distribution peaking at 40.9 µm and at 28.2 µm, respectively. 

In conclusion, rheology modification (by PVA) is found to be the most effective strategy to stabilize O/W emulsion systems for the PUa MCs’ synthesis by interfacial polymerization. The increased viscosity of the continuous phase, achieved with PVA at 2 wt% in the aqueous phase of the emulsion, is an effective strategy to reduce the gravitational separation phenomenon (droplet sedimentation) and to finely control the size of the final MCs, resulting in relatively small MCs with 46 wt% of encapsulated isocyanate that are, therefore, acceptable for the envisaged application as solid crosslinkers for mono-component adhesives. 

## Figures and Tables

**Table 1:** Microcapsules’ acronyms and emulsion composition.

Acronym	Water Phase	Oil Phase	Emulsion Stabilizer (wt% Added to the W Phase)
GA_MCs	Water + Emulsion stabilizer	Ongronat^®^2500 + Desmodur^®^ I (IPDI)	GA (5%)
DC_MCs			DC193 (4%)
GA_DC_MCs			GA (4%) DC193 (1%)
PVA_MCs			PVA (2%)
GA_PVA_MCs			GA (1.3%) PVA (2%)

**Table 2:** MCs’ diameter, average shell thickness (s) and S/D ratio.

MCs’ Acronym	D, MC’s Diameter (Peak Max, Mode) (μm)	S, Av. Shell Thickness (μm)	S/D (Ratio)
GA_MCs	92.7	3.4 ± 1.3	0.04 ± 0.01
DC_MCs	80.2	6.1 ± 3.3	0.08 ± 0.04
GA_DC_MCs	28.2	1.8 ± 0.3	0.06 ± 0.01
PVA_MCs	40.9	4.1 ± 3.7	0.10 ± 0.10
GA_PVA_MCs	65.3	1.9 ± 0.9	0.03 ± 0.01

**Table 3:** Mass loss (%) of encapsulated IPDI (by TGA), relative encapsulation Yield (Y) calculated using the FTIR spectra obtained after the synthesis and 3 months later. The Y obtained after the synthesis was calculated using (Equation (2). Y_3_ is given as a percentage of Yi.

MCs’ Acronym	Mass Loss (%) from TGA (Encapsulated Isocyanate)	Initial Relative Encapsulation Yield (Yi)	Y_3_ (%) 3 Months after the Synthesis
GA_MCs	31.2 wt%	14	50.3
DC_MCs	30.6 wt%	13.7	62.3
GA_DC_MCs	32.9 wt%	14.3	34.2
PVA_MCs	46 wt%	15.3	45.8
GA_PVA_MCs	46.3 wt%	16.1	53.3

## Data Availability

The data presented in this study are available in the present article and in the related Supplementary Materials.

## Supplementary Materials

The following supporting information can be downloaded at: https://www.mdpi.com/article/10.3390/polym15020403/s1, Scheme S1: Schematic representation of the emulsion’s classification. Table S1: Stabilizers used for the O/W emulsion stabilization and respective molecular structure, HLB and molecular weight. Scheme S2: Schematic representation of the microcapsules’ synthesis process. Table S2: Optical microscopy photographs of all the emulsions tested after 10 min of emulsification and after the following 15 min, under mechanical stirring at 400 rpm (dynamic conditions). Scheme S3: Schematic representation of the sedimentation phenomenon observed in a polydisperse emulsion system. Figure S1: Optical microscopy photographs of the emulsion that suffered less sedimentation (GA_PVA_MCs emulsions) and that of the emulsion stabilized with DC193. Figure S2: SEM photomicrograph of the MCs obtained using GA, PVA and the combination of both for the emulsion stabilization. The MCs were synthetized after the respective emulsions were let at rest for 72 h, after the emulsification (static conditions). Figure S3: Optical microscopy photographs of the DC_MCs’ emulsion during the first 25 min of the synthesis (dynamic conditions). Table S3: EDS atomic concentrations of the DC_MCs for C, N, O and Si atoms. Figure S4. Corresponding areas of the EDS analysis. Figure S5. Thermograms of the MCs’ shell.
