# Supplementary material for: Emulsion Stabilization Strategies for Tailored Isocyanate Microcapsules
Source: Polymers (Basel). 2023 Jan 12;15(2):403. doi: 10.3390/polym15020403 (PMC9865233; doi:10.3390/polym15020403)
Supplement: Supplementary file 1 [file polymers-15-00403-s001.zip › polymers-2122161-supplementary.pdf]

Supplementary File

Emulsion Stabilization Strategies for Tailored Isocyanate Microcapsules

Mónica V. Loureiro [1], António Mariquito [1], Mário Vale [1], João C. Bordado [1], Isabel Pinho [2] and Ana C. Marques [1,*]

[1] CERENA-Centro de Recursos Naturais e Ambiente, Departamento de Engenharia Química, Instituto Superior Técnico, Universidade de Lisboa, Avenida Rovisco Pais, 1049-001 Lisbon, Portugal

[2] CIPADE-Indústria e Investigação de Produtos Adesivos, SA. Av. Primeiro de Maio 121, 3700-227 São João da Madeira, Portugal

* Correspondence: ana.marques@tecnico.ulisboa.pt

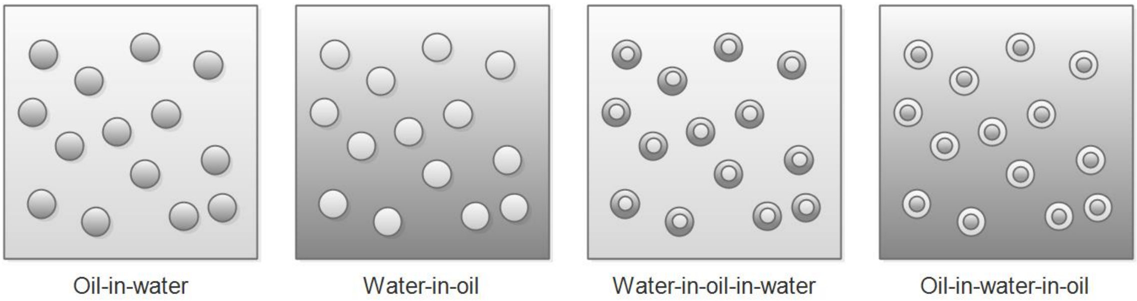

Scheme S1. Schematic representation of the emulsion's classification.

Table S1. Stabilizers used for the O/W emulsion stabilization and respective molecular structure, HLB and molecular weight.

Emulsion Stabilizers	Molecular Structure	Type of Stabilizer	HLB	Molecular Weight (Da.)
**GA**	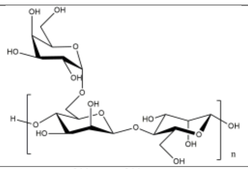	Polysaccharide emulsifier	n.a.	250 000
**DC193**	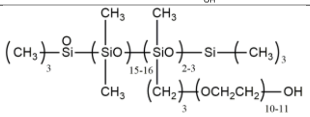	Silicone surfactant	12	3 200
**PVA**	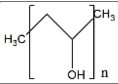	Rheology modifier	n.a.	57 000 -66 000

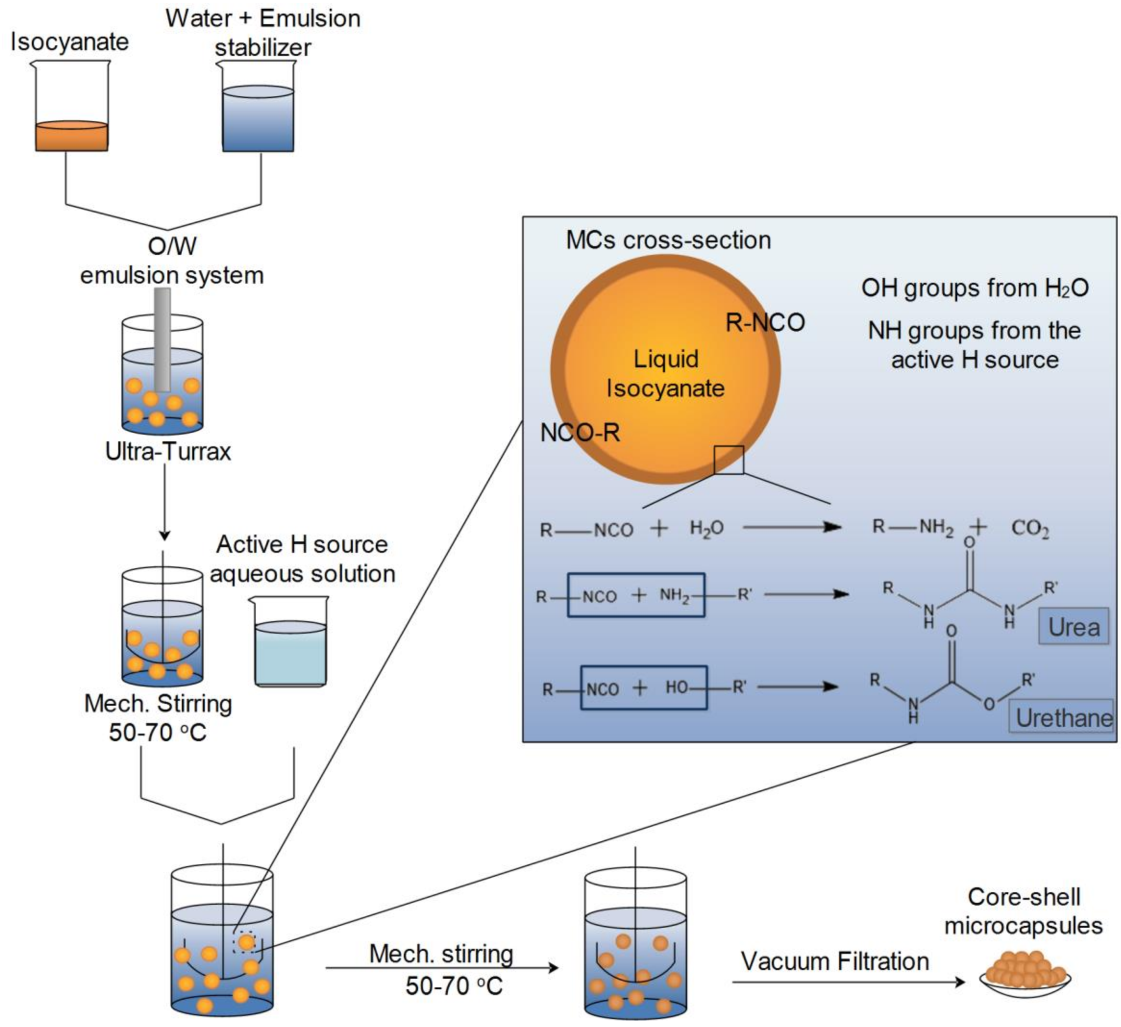

Scheme S2. Schematic representation of the microcapsules' synthesis process.

Table S2. Optical microscopy photographs of all the emulsions tested. Left: after 10 minutes of emulsification at 3200 rpm using the Ultra-Turrax; Right: after the following 15 minutes under mechanical stirring at 400 rpm (dynamic conditions)

**Emulsion stabilizer**	**Stabilizer (wt% added to the W phase)**	**Ultra-Turrax emulsification**	**Mechanical Agitation**
Gum arabic (GA)	5%	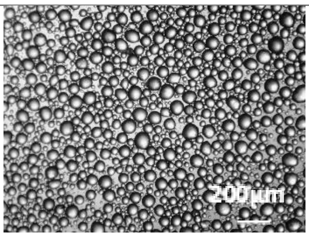	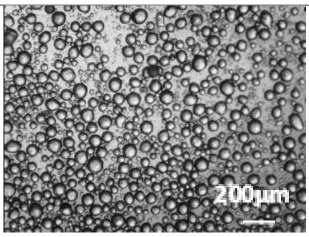
DC193	2.5%	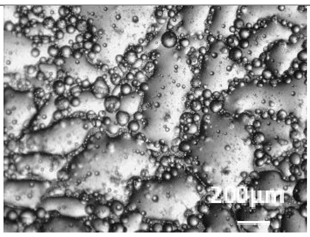	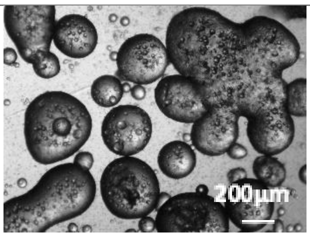
	4%	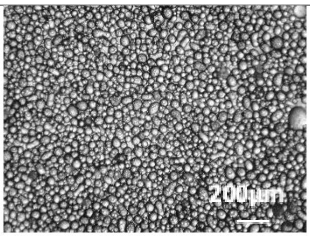	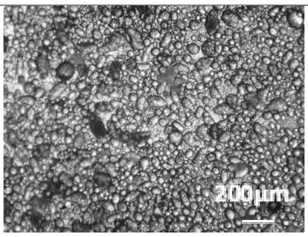
	5%	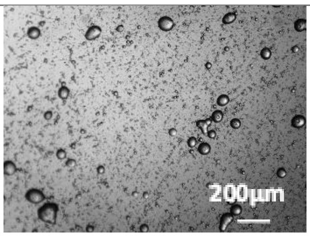	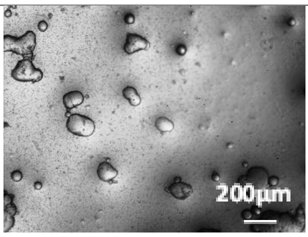
	6%	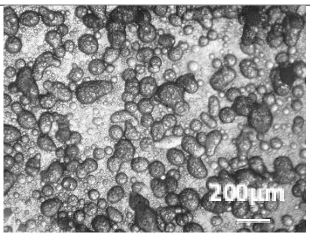	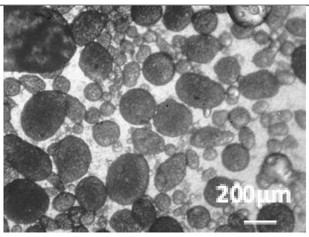
DC193 and GA	2% (DC193) and 2.5% (GA)	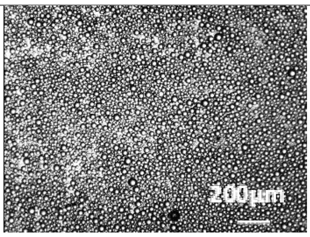	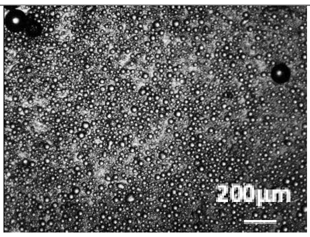

	4% (DC193) and 1% (GA)	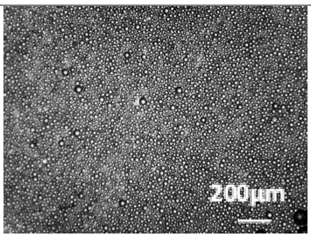	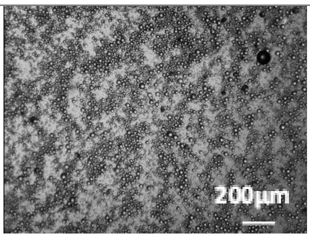
Polyvinyl alcohol (PVA)	2%	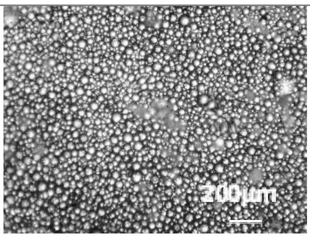	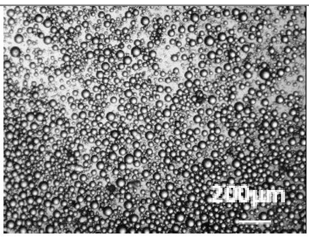
	3%	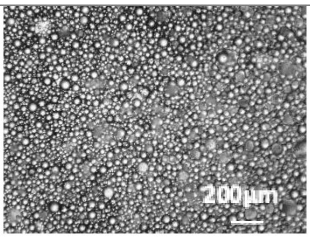	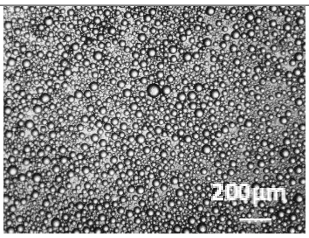
PVA and GA	2% (PVA) and 1.30% (GA)	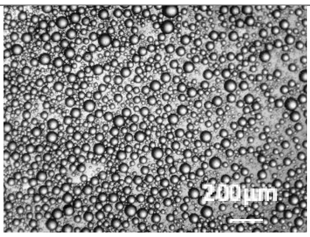	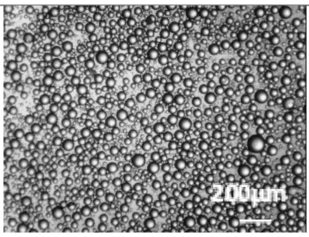
	2wt% (PVA) and 2.5% (GA)	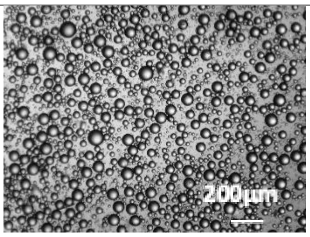	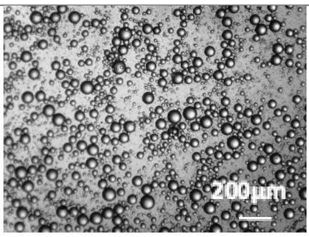
SPAN®20	2.5%	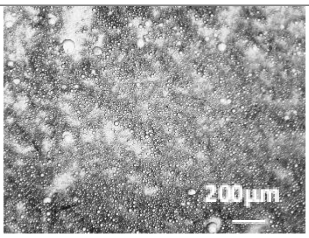	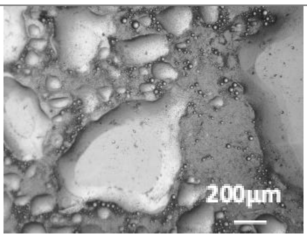
	4%	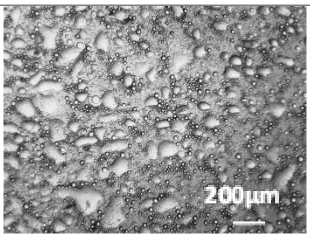	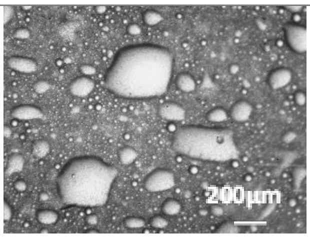

	5%	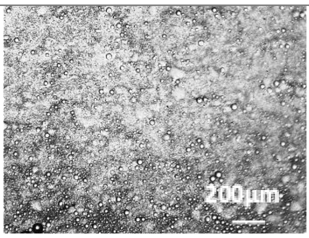	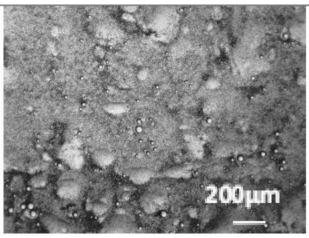
	6%	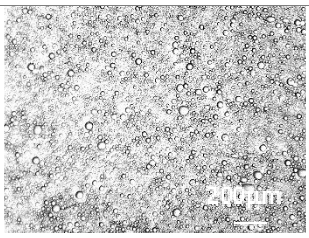	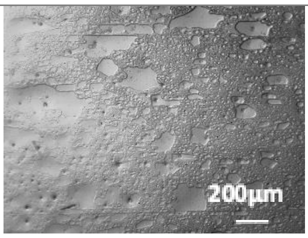
Tween®85	2.5%	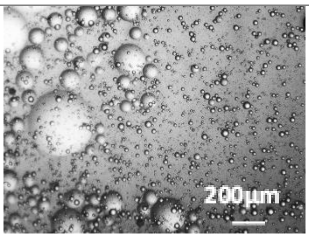	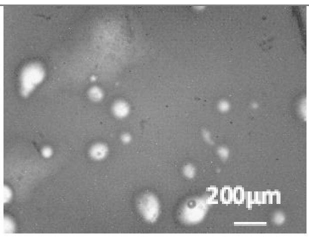
	4%	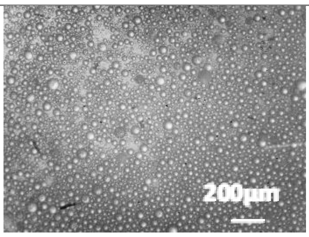	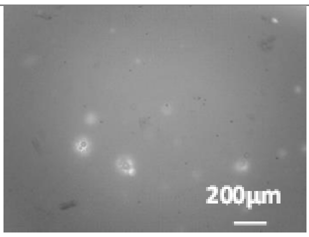
	5%	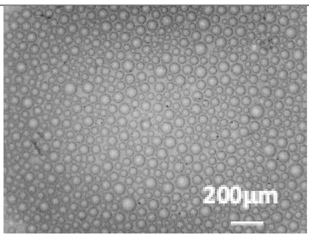	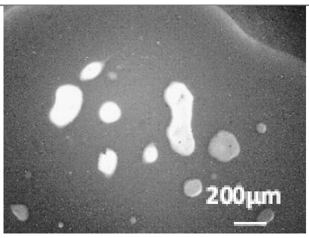
Pluronic® P123	2.5%	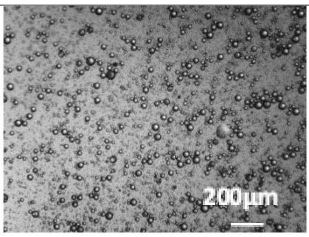	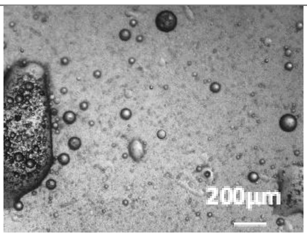
	4%	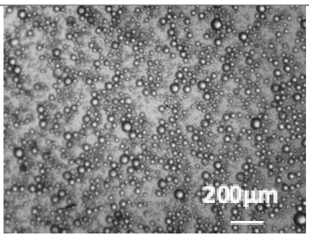	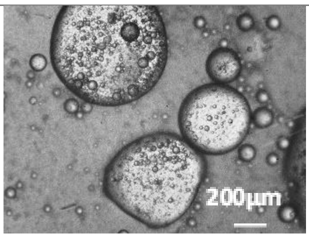

	5%	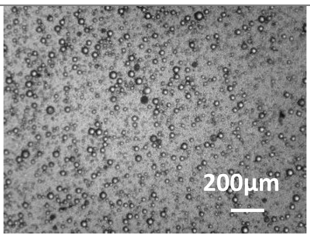	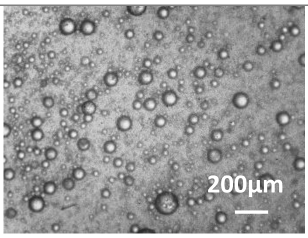
	6%	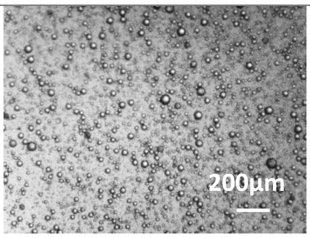	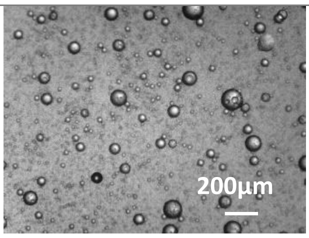
Pluronic® P123 and GA	2% (Pluronic P123) and 2.5% (GA)	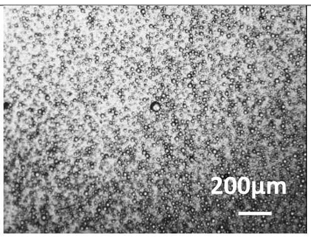	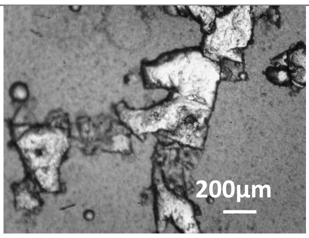
	1% (Pluronic P123) and 4% (GA)	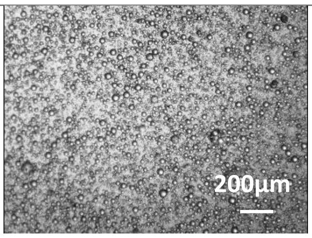	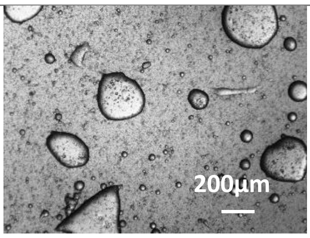

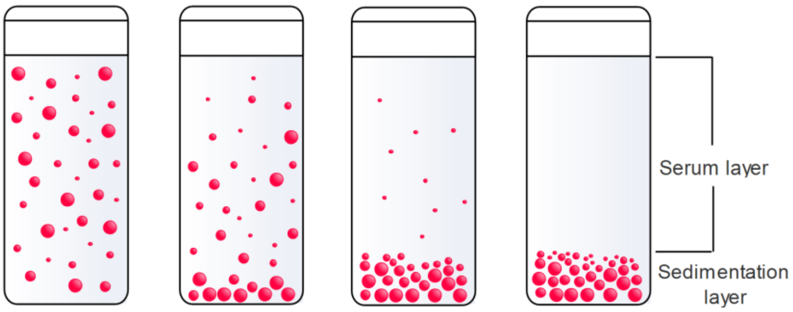

Scheme S3. Schematic representation of the sedimentation phenomenon observed in a polydisperse emulsion system.

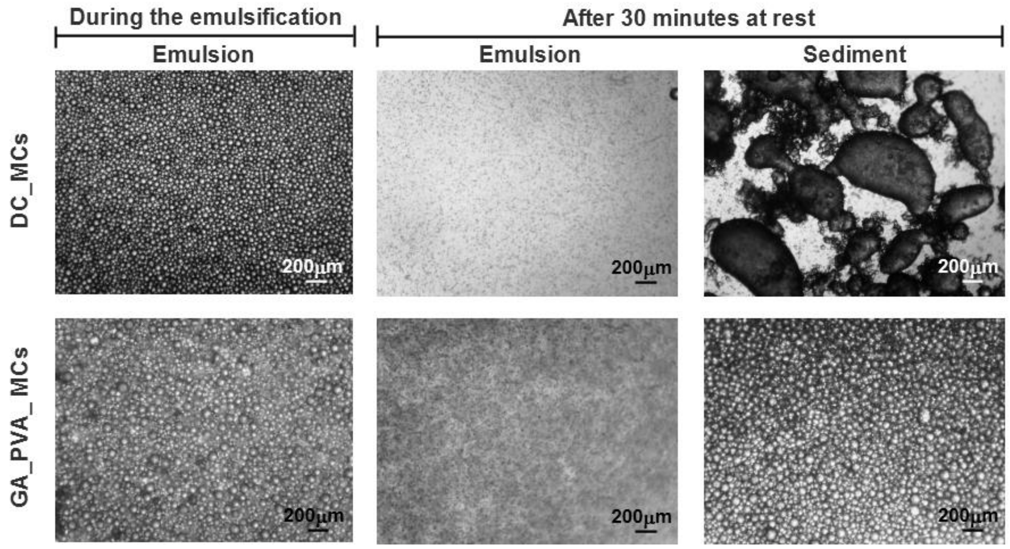

Figure S1. Optical microscopy photographs of the emulsion that suffered less sedimentation (GA_PVA_MCs emulsions) and that of the emulsion stabilized with DC193. At the left when under emulsification and the right the respective emulsion and sediment portions when at rest for 30 minutes (static conditions).

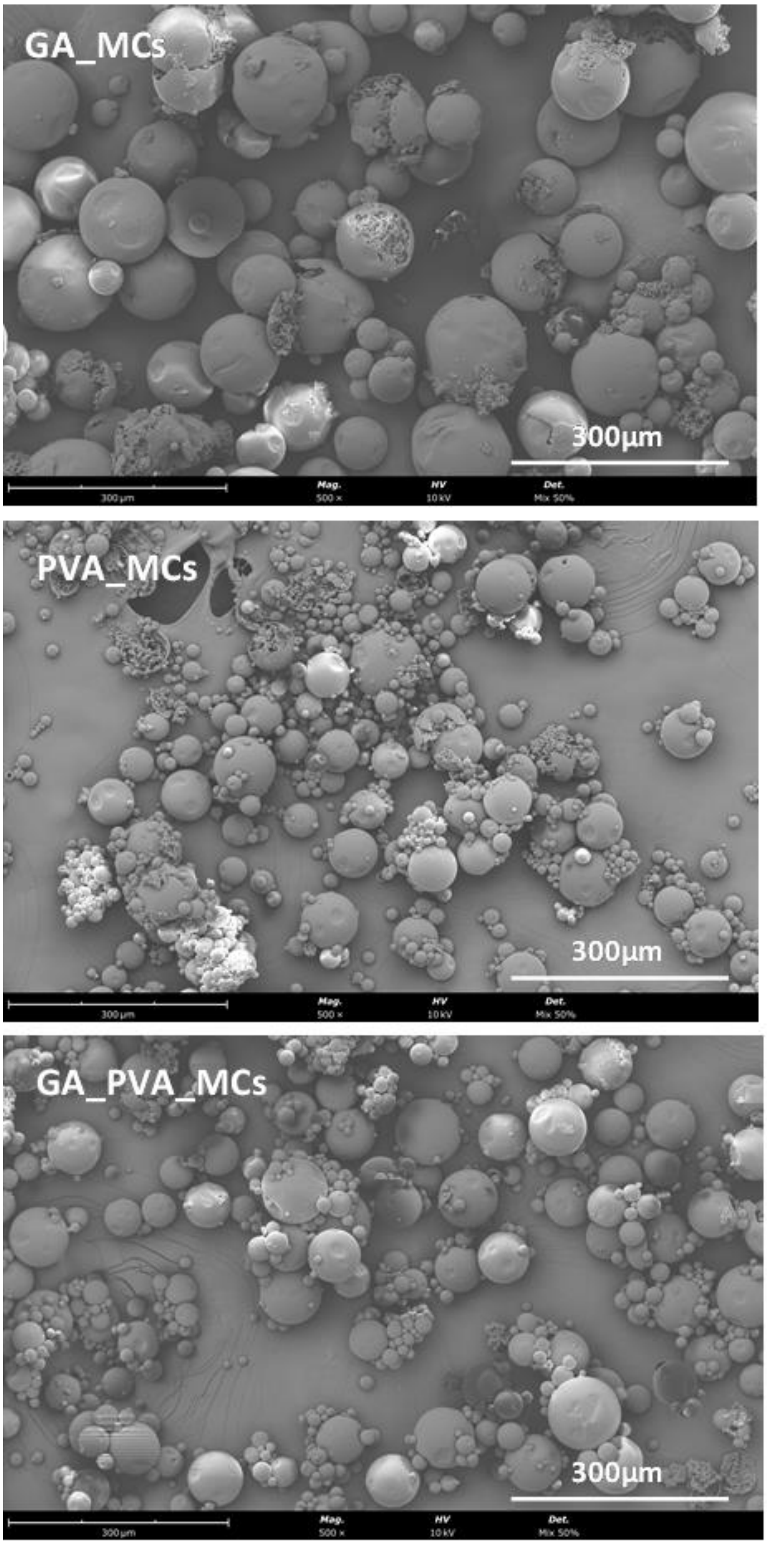

Figure S2. SEM photomicrograph of the MCs obtained using GA, PVA and the combination of both for the emulsion stabilization. The MCs were synthetized after the respective emulsions were let at rest for 72h, after the emulsification (static conditions).

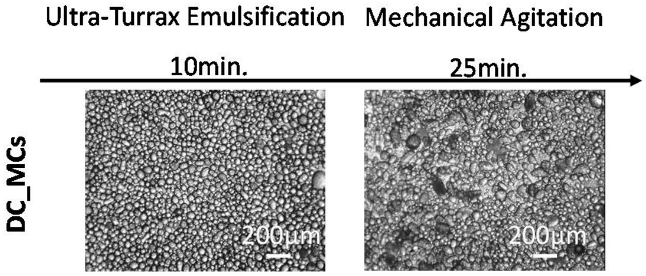

Figure S3. Optical microscopy photographs of the DC_MCs' emulsion during the first 25 minutes of the synthesis. Left: after 10 minutes of emulsification at 3200 rpm using the Ultra-Turrax; Right: after the following 15 minutes under mechanical stirring at 400 rpm (dynamic conditions).

Table S3. EDS atomic concentrations of the DC_MCs for C, N, O and Si atoms

Analysis	Atomic concentration			
	C	N	O	Si
**Area 1**	61.782	26.95	10.24	1.03
**Area 2**	61.084	27.49	10.73	0.70
**Area 3**	70.502	19.58	6.68	3.24

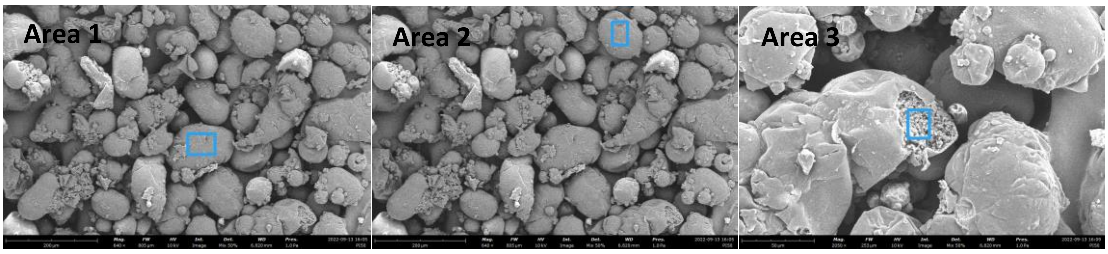

Figure S4. Corresponding areas of the EDS analysis

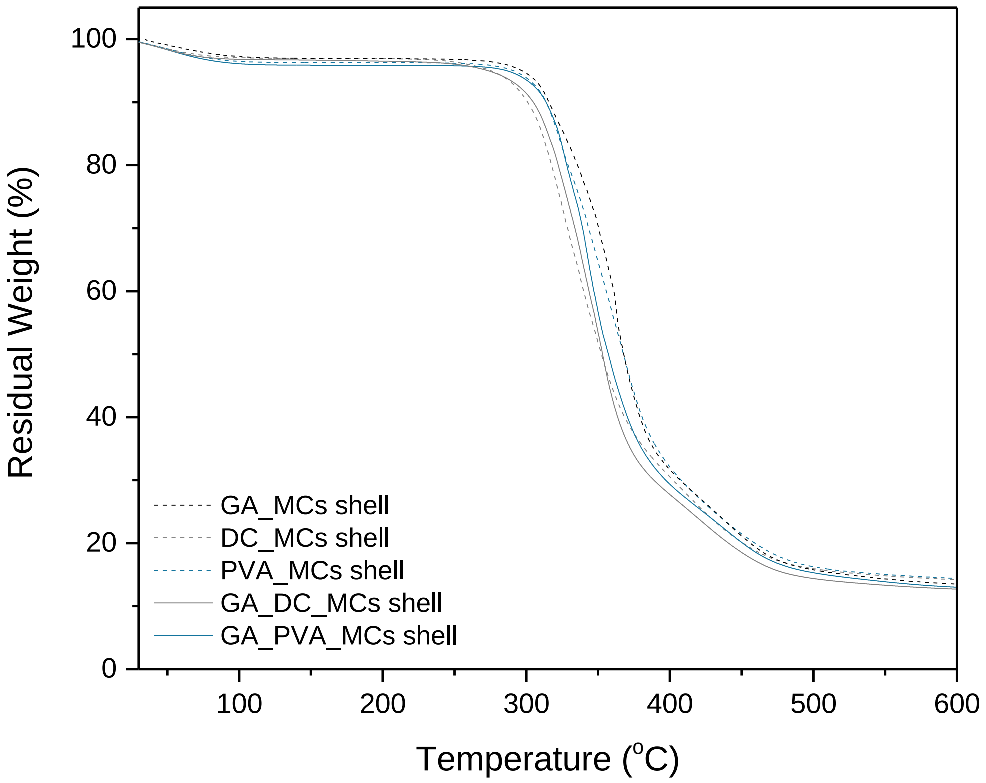

Figure S5. Thermograms of the MCs' shell.
